# Histotripsy for portal vein tumor thrombus in a patient with hepatocellular carcinoma: a case report

**DOI:** 10.3389/fonc.2025.1721814

**Published:** 2025-12-02

**Authors:** Kevin Burns, Tiffany M. Juarez

**Affiliations:** 1 Department of Interventional Radiology, Providence Mission Hospital, Mission Viejo, CA, United States; 2 Leonard Cancer Institute, Providence Mission Hospital, Mission Viejo, CA, United States

**Keywords:** portal vein tumor thrombus, hepatocellular carcinoma, histotripsy, locoregional, salvage therapy

## Abstract

The presence of portal vein tumor thrombus (PVTT) in patients with hepatocellular carcinoma (HCC) carries a poor prognosis and significantly reduces effective treatment options. Here, we present a case of a 76-year-old male with Barcelona Clinic Liver Cancer stage C (BCLC C) HCC and extensive PVTT who was treated with histotripsy having progressed through both locoregional and systemic therapy. Histotripsy produced rapid cavitation of the tumor thrombus eliciting an imaging response in the process and liver regeneration in the treated segment. This case highlights the feasibility of noninvasive histotripsy as a salvage therapy for PVTT from HCC.

## 1 Introduction

The presence of portal vein tumor thrombus (PVTT) in patients with hepatocellular carcinoma (HCC) is a poor prognostic indicator placing these patients squarely into the advanced stage category of the BCLC staging system (BCLC C) where treatment options are limited to systemic drug therapy according to the BCLC staging system and treatment recommendations (1). The use of locoregional therapies for these patients is therefore limited and somewhat controversial due to the lack of robust clinical data (2).

Histotripsy, a noninvasive focused ultrasound therapy that induces mechanical tissue destruction via cavitation, has shown early promise in treating unresectable hepatic tumors. First, the feasibility THERESA trial demonstrated the short-term efficacy and safety of histotripsy for liver tumors (3) and then the multi-center, pivotal #HOPE4LIVER trial reported promising clinical outcomes, such as 90% local tumor control at 1-year (4–6). Histotripsy’s favorable safety profile was confirmed in an international analysis involving 230 patients in which only 5% of patients experienced complications of any grade (7). Finally, another study conducted on patients with liver metastases from pancreas cancer again demonstrated the positive clinical impact of histotripsy (8).

Histotripsy offers a compelling technical advantage over other locoregional therapies through its precision in targeting tumors near collagen-rich structures such as bile ducts and blood vessels, maintaining treatment efficacy in complex anatomical areas. Here histotripsy was successfully used to treat PVTT in a patient with HCC refractory to both Y90 radioembolization and systemic therapy.

## 2 Case report

**Figure 1:**
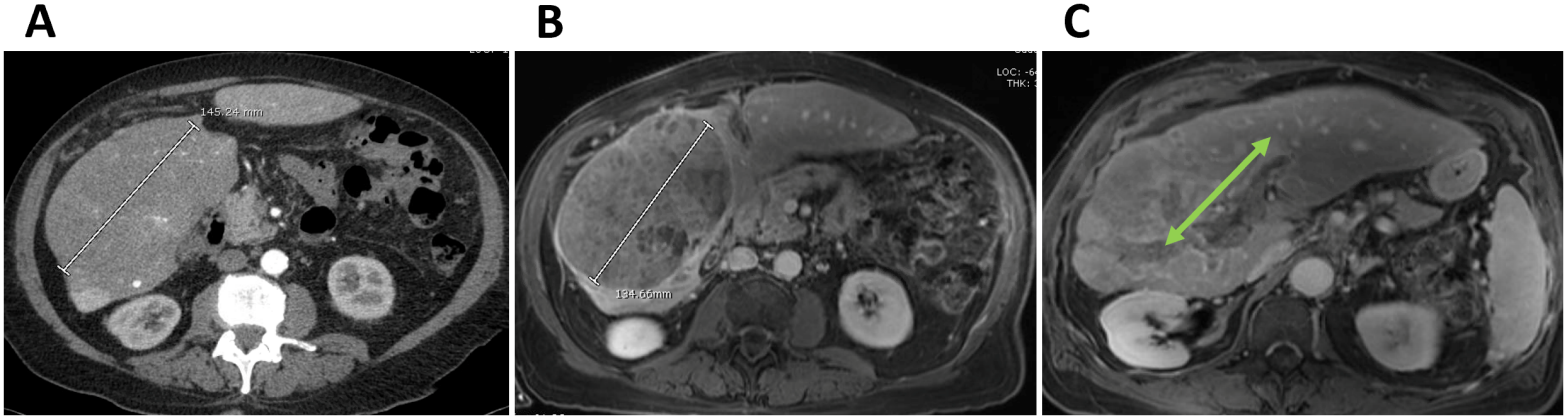
**(A)** Axial contrast-enhanced CT scan of liver showing the extent of the large HCC throughout the right lobe at diagnosis (AFP 2,838 ng/dL). **(B, C)** Axial contrast-enhanced T1-weighted MR images after initiation of systemic therapy with atezolizumab-bevacizumab showing stable disease **(B)** and tumor extension into portal vein branches (arrow) **(C)**.

**Figure 2:**
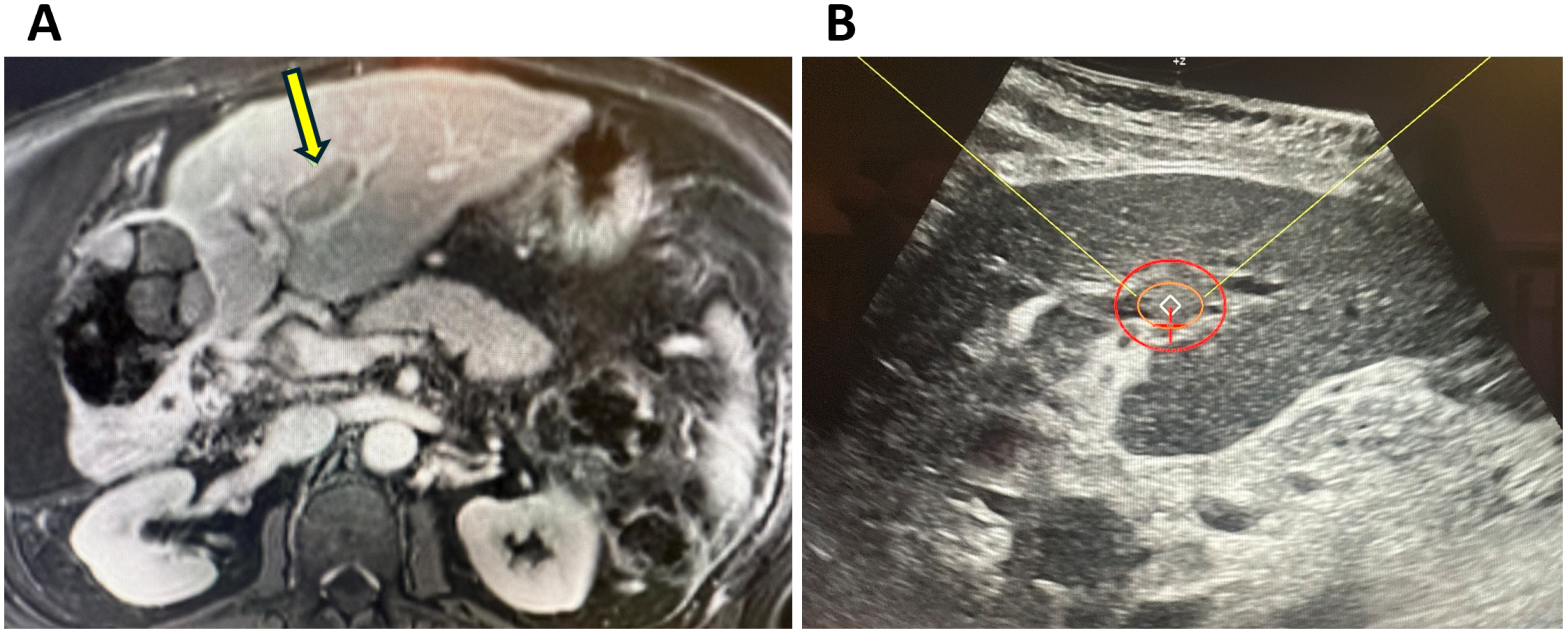
**(A)** Axial contrast-enhanced T1-weighted MR image after Y90 therapy showing the dominant HCC in the right lobe without any enhancement, consistent with near complete response according to mRECIST. The yellow arrow points to the portal vein tumor thrombus before targeting by histotripsy. **(B)** Preoperative ultrasound of the portal vein tumor thrombus being targeted by histotripsy, with the planning treatment volume (orange circle) surrounded by the treatment margin (red circle).

A 77-year-old male with chronic hepatitis C and cirrhosis (Child-Pugh A) presented with a 14 cm right hepatic lobe mass and PVTT extending into the main and left portal veins in July 2023 (**Figure 1A**). In accordance with the BCLC treatment guidelines for HCC-45, he was initially treated with atezolizumab-bevacizumab combination systemic therapy. Magnetic resonance (MR) imaging in December 2023 showed stable disease and tumor extension into portal vein branches (**Figures 2B, C**). Two sessions of Y90 radioembolization were administered (April 2024 and May 2024) substantially decreasing the size of the dominant tumor to 7 × 5 cm without affecting the PVTT. Upon disease progression in July 2024, he was switched to tremelimumab/durvalumab but discontinued after one cycle due to colitis, which prompted another switch, this time, to sorafenib in December 2024.

**Figure 3:**
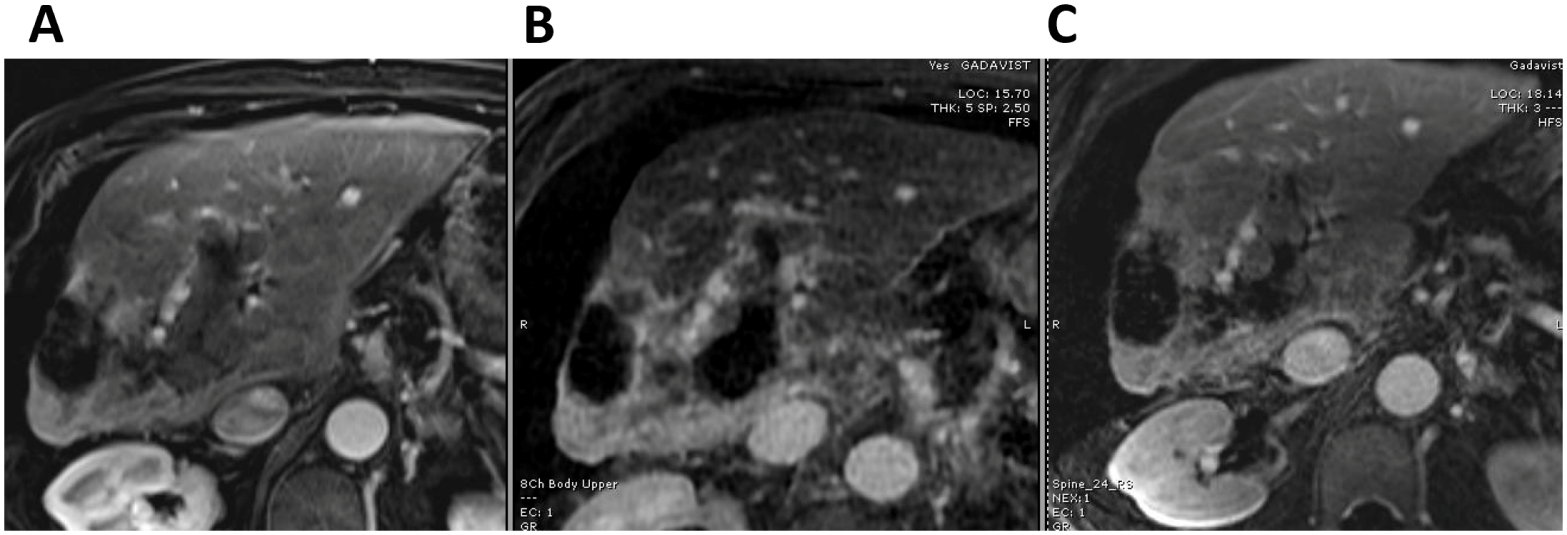
Axial contrast-enhanced T1-weighted MR images of the liver showing the treatment effect of histotripsy in the portal vein tumor thrombus prior to histotripsy **(A)** and six-weeks **(B)** and four months **(C)** post histotripsy showing the degree of cavitation of the portal vein tumor thrombus due to histotripsy.

During a tumor board discussion, histotripsy was recommended as a treatment option to specifically target the PVTT. Therefore, in January 2025, the patient underwent histotripsy with the goal of targeting the left portal vein tumor thrombus (**Figure 2**). On the day of the procedure, the patient presented with elevated bilirubin (1.3 mg/dL) and alkaline phosphatase (175 U/L), whereas ALT and AST were within normal range. Histotripsy was performed with the Edison System (HistoSonics, Plymouth, Minnesota) as previously described (9). The procedure was performed under general anesthesia with left-sided double lumen intubation under the direction of an experienced anesthesiologist. Tidal volume and PEEP were adjusted as needed to limit breathing motion and limit the presence of lung tissue in the ultrasound beam path. Targeting of the two lesions was conducted with a handheld ultrasound device to optimize the positioning and angulation needed with the treatment transducer (treatment head). The coupler with de-ionized water and patented membrane was added and targeting done with the space mouse and Edison controls. In addition to ensuring that the tumor was adequately covered, a “surgical” margin was included to account for breathing motion and microscopic extension, and care was made to avoid overlap of the planned treatment volume (PTV) with normal organs at risk including lung, bowel, and stomach. Once targeting was optimized, the Edison System produced *in vitro* test pulses to ensure adequate histotripsy could be achieved at 7 test locations and then autonomous delivery of histotripsy ensued using a robotic treatment arm with exquisite precision. Two overlapping treatments were delivered to the tumor thrombus, with an average voltage of 46.0% for 5.3 minutes of treatment time to the 2.65 cm PTV maximum diameter and an average voltage of 59.0% for 11.2 minutes to the 2.8 cm PTV maximum diameter. Once treatment was completed, the coupler with the patented membrane was removed and the patient was removed from anesthesia. A CT scan was performed within 24 hours to evaluate the immediate technical efficacy of the procedure as defined in the Hope4Liver study (6). The patient tolerated the procedure well without any peri-procedural complications and was discharged the same day. Follow-up MR imaging at the end of January 2025 showed observable cavitation of the treated thrombus within the portal vein (**Figures 3A, B**).

A second histotripsy procedure was performed in February 2025, this time delivering three overlapping treatments (PTV of 3.0, 3.2, and 2.0 cm corresponding to a treatment time of 7.3, 15 and 4.2 minutes, respectively) to the tumor thrombus located within the right and main portal veins. The patient again tolerated the procedure well without any toxicities. The treated thrombus continued to collapse on the subsequent MRI in June 2025 (**Figure 3C**) and the liver parenchyma surrounding the treated portal vein appeared to regenerate. The alpha-fetoprotein (AFP) serum level concomitantly decreased from 140 ng/L before the second histotripsy to 25 ng/L (normal level 0.0 - 8.4 ng/mL). Although the portal vein segments that were treated by histotripsy did not recanalize, the pre-existing cavernous transformation of the portal venous system supplying the liver parenchyma remained patent.

**Figure 4:**
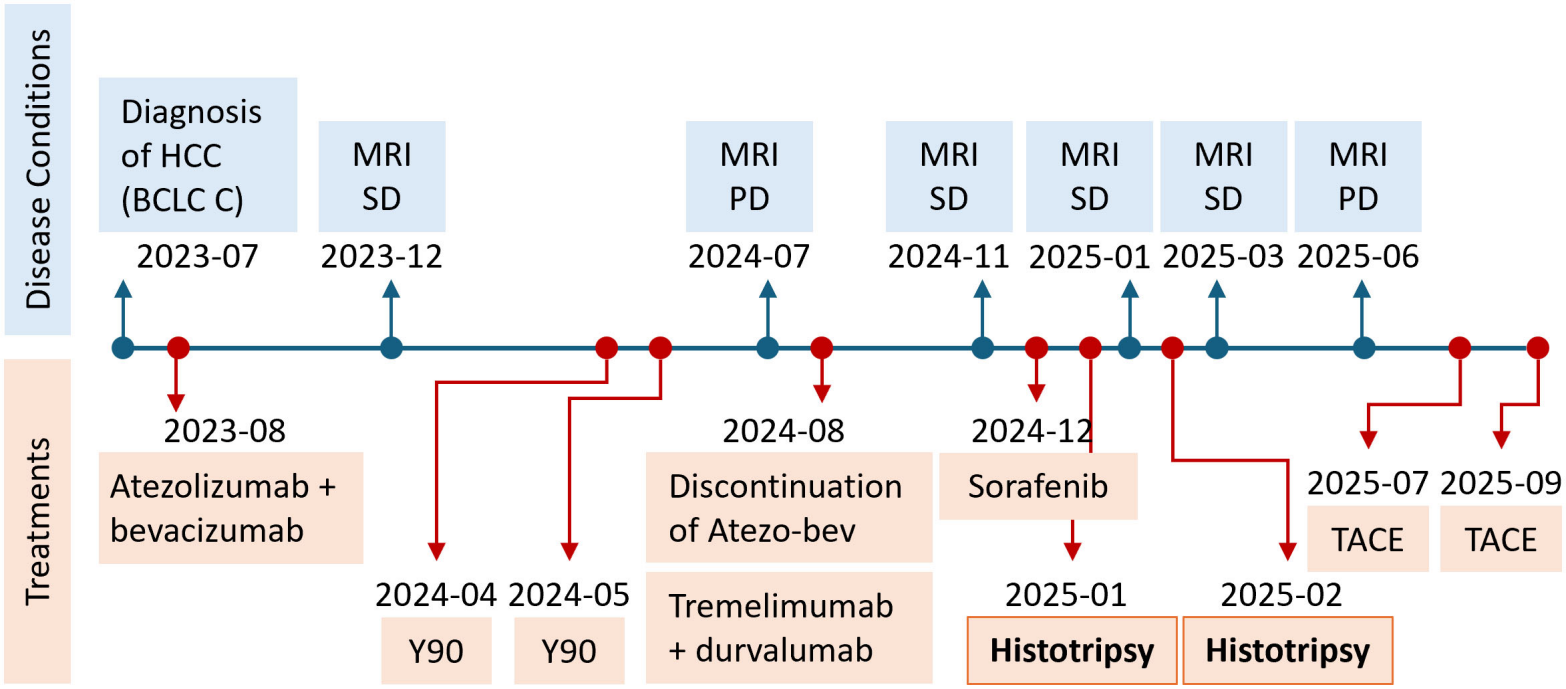
A schematic time course of the entire case.

Although the patient continued to receive sorafenib during and after the sessions of histotripsy, the restaging MRI in June 2025 demonstrated interval disease progression with the appearance of a new distinct liver lesion, and mild to moderate ascites that did not require any paracentesis. The patient was started on regorafenib before receiving two cycles of transarterial chemoembolization (TACE) to the caudate branch in July 2025 and to the right hepatic lobe in September 2025. Note that the previously treated PVTT remains stable without any evidence of progression, At the most recent follow-up evaluation in September 2025, the patient is clinically stable without any sign of liver decompensation, with preserved liver function (Child-Pugh A), ECOG 0, and no sign of hepatic encephalopathy. A timeline of the patient’s treatment course is shown in **Figure 4**.

## 3 Discussion

Macrovascular invasion or the direct extension of tumor into the portal venous system is one of the worst prognostic indicators of patients with HCC as determined by the BCLC staging system and is notoriously difficult to treat. It is largely responsible for the worsening of liver function resulting in liver failure and death that is typical for patients with advanced HCC. According to the BCLC treatment guidelines, HCC patients with PVTT are considered advanced (BCLC C) and should be treated with systemic drug therapy (1). A role for loco-regional therapy in this patient population is questionable. However, this case demonstrates the possible utility of a new noninvasive locoregional image-guided therapeutic option, namely histotripsy, to specifically target the PVTT. The rapid destruction of the tumor thrombus and subsequent regeneration of adjacent liver tissue, as shown in our case, suggest a potential role for histotripsy in restoring vascular patency which could prevent liver decompensation and possibly reverse liver dysfunction (10).

Importantly, the cavernous transformation of the portal venous system, which provided critical blood flow to the liver parenchyma despite the tumor thrombus in the left and main portal veins, was left unaffected by histotripsy, demonstrating the unique ability of histotripsy to destroy tissue through mechanical cavitation rather than thermal deposition in an extremely precise manner under direct imaging guidance while leaving vascular structures intact. Despite the evident technical success of the procedure, as demonstrated by the cavitation of the tumor thrombus within the portal vein, the main portal vein did not recanalize, yet the liver perfusion remained unchanged without any evidence of ischemia or infarction.

The technical success of histotripsy as seen in this patient may allow stage migration of patients with chronic and extensive PVTT from BCLC C to B (intermediate) and possibly even A (early-stage HCC) thereby allowing patients to receive treatments that would not be available under the BCLC staging system and treatment guidelines.

Although case reports are limited by their inability to generalize findings across a broader patient population due to the lack of meaningful statistical analysis, absence of control group, and potential confounding factors, the promising results observed with histotripsy in this case suggest that, if validated through well-controlled studies, such an approach could improve outcomes for patients with advanced HCC and PVTT.

## 4 Conclusion

Histotripsy enabled safe and effective noninvasive destruction of tumor thrombus within the portal vein in a patient with advanced HCC. This new approach using histotripsy may expand treatment options for patients with limited therapeutic alternatives.

## Data Availability

The raw data supporting the conclusions of this article will be made available by the authors, without undue reservation.
